# Discovery and Characterization of Phage Display-Derived Human Monoclonal Antibodies against RSV F Glycoprotein

**DOI:** 10.1371/journal.pone.0156798

**Published:** 2016-06-03

**Authors:** Zhifeng Chen, Lan Zhang, Aimin Tang, Cheryl Callahan, Pavlo Pristatsky, Ryan Swoyer, Pedro Cejas, Debbie Nahas, Jennifer Galli, Scott Cosmi, Daniel DiStefano, Van M. Hoang, Andrew Bett, Danilo Casimiro, Kalpit A. Vora

**Affiliations:** 1 Department of Infectious Diseases and Vaccines Research, Merck Research Laboratories, Merck & Co., Inc., West Point, PA, United States of America; 2 Vaccine Bioprocess Research and Development, Merck Research Laboratories, Merck & Co., Inc., West Point, PA, United States of America; 3 Eurofins Lancaster Laboratories Professional Scientific Services, Lancaster, PA, United States of America; Imperial College London, UNITED KINGDOM

## Abstract

Respiratory syncytial virus (RSV) is a leading cause of lower respiratory tract infection in infants, the elderly and in immunosuppressed populations. The vast majority of neutralizing antibodies isolated from human subjects target the RSV fusion (F) glycoprotein, making it an attractive target for the development of vaccines and therapeutic antibodies. Currently, Synagis^®^ (palivizumab) is the only FDA approved antibody drug for the prevention of RSV infection, and there is a great need for more effective vaccines and therapeutics. Phage display is a powerful tool in antibody discovery with the advantage that it does not require samples from immunized subjects. In this study, Morphosys HuCAL GOLD^®^ phage libraries were used for panning against RSV prefusion and postfusion F proteins. Panels of human monoclonal antibodies (mAbs) against RSV F protein were discovered following phage library panning and characterized. Antibodies binding specifically to prefusion or postfusion F proteins and those binding both conformations were identified. 3B1 is a prototypic postfusion F specific antibody while 2E1 is a prototypic prefusion F specific antibody. 2E1 is a potent broadly neutralizing antibody against both RSV A and B strains. Epitope mapping experiments identified a conformational epitope spanning across three discontinuous sections of the RSV F protein, as well as critical residues for antibody interaction.

## Introduction

Human respiratory syncytial virus (RSV) is an enveloped virus of the family *Paramyxoviridae* with a single-stranded non-segmented negative-sense RNA genome. RSV is the most important cause of acute lower respiratory tract infections (ALRI) in infants worldwide, which can lead to bronchiolitis and pneumonia [1, 2]. In the United States, RSV infects nearly all children by two years of age [3]. RSV is also identified as a leading cause of ALRI among the elderly and immuno-compromised populations worldwide [4, 5]. Passive immunotherapy with a monoclonal antibody palivizumab (Synagis®, Astra-Zenaca) for the prevention of serious lower respiratory tract disease caused by RSV is available for high-risk infants. However it has only modest efficacy and the dose used for infants makes it cost-prohibitive for use in the adult population [6]. Efficacious vaccines or more potent antibodies are needed for protection of all children as well as adults from RSV infection.

RSV encodes 11 proteins, two of which (a type I fusion protein F and attachment protein G) give rise to neutralizing antibodies. Out of these two RSV glycoproteins, the F protein is the target of palivizumab and the major target of neutralizing antibodies in human sera [7–9]. Two antigenic groupings of human RSV exist (A and B). These groupings are based on reactivity to antibodies and amino acid sequence comparisons, and primarily focused on the sequence of the RSV G protein. RSV F is well conserved among clinical isolates and between the RSV-A and RSV-B antigenic subgroups. Therefore, F protein appears to be an attractive target for vaccines and therapeutic antibodies. F protein exists in two distinct conformations: the metastable prefusion conformation and the stable postfusion conformation [10, 11]. Although targets for neutralizing monoclonal antibodies exist on both the prefusion and the postfusion conformations of F protein, characterization of the natural immune response to RSV infection revealed that most RSV-neutralizing antibodies elicited in humans target the prefusion conformation of the F protein [8, 9].

Multiple neutralizing epitopes on the RSV F protein have been identified, including antigenic site II on both prefusion and postfusion F where palivizumab binds [12]. Recently, extremely potent antibodies that specifically target the prefusion F protein have been identified from human peripheral blood, including D25 which reacts to antigenic site 0 [11] and MPE8 which binds to antigenic site III [13].

We sought to find RSV F specific antibodies from a phage display library as an alternative approach to identifying potent monoclonal antibodies. Phage display technology was first invented by George Smith in 1985 [14], and was developed largely in the 1990s [15–17]. The construction of phage display libraries does not require immunized subjects, and the libraries can even be fully synthetic [18]. It is a powerful, versatile and time-saving platform. Several monoclonal antibodies (mAbs) have been discovered through this platform [19, 20], including mAbs already approved by FDA and currently on market[21].

The Morphosys HuCAL GOLD^®^ library is a synthetic, fully human antibody library containing 1.2x10^10^ different functional human antibody genes. This extremely large library of antibody molecules permits the recognition of a large number of foreign molecules. Thus, it is an excellent choice for the *in vitro* discovery of specific human mAbs for target validation and therapeutic uses [22, 23].

In this study, we used Morphosys HuCAL GOLD^®^ phage libraries for panning against pre- and postfusion RSV F proteins. We have discovered and characterized panels of human mAbs that specifically react against pre- and/or postfusion F proteins. The human mAbs discovered in this study can be used as critical reagents in antigen detection, identification and characterization, to facilitate development of RSV vaccines and therapeutics.

## Results

### Antibodies against RSV prefusion and postfusion F proteins were identified from Morphosys HuCAL GOLD ^®^ phage display libraries

**Fig 1:**
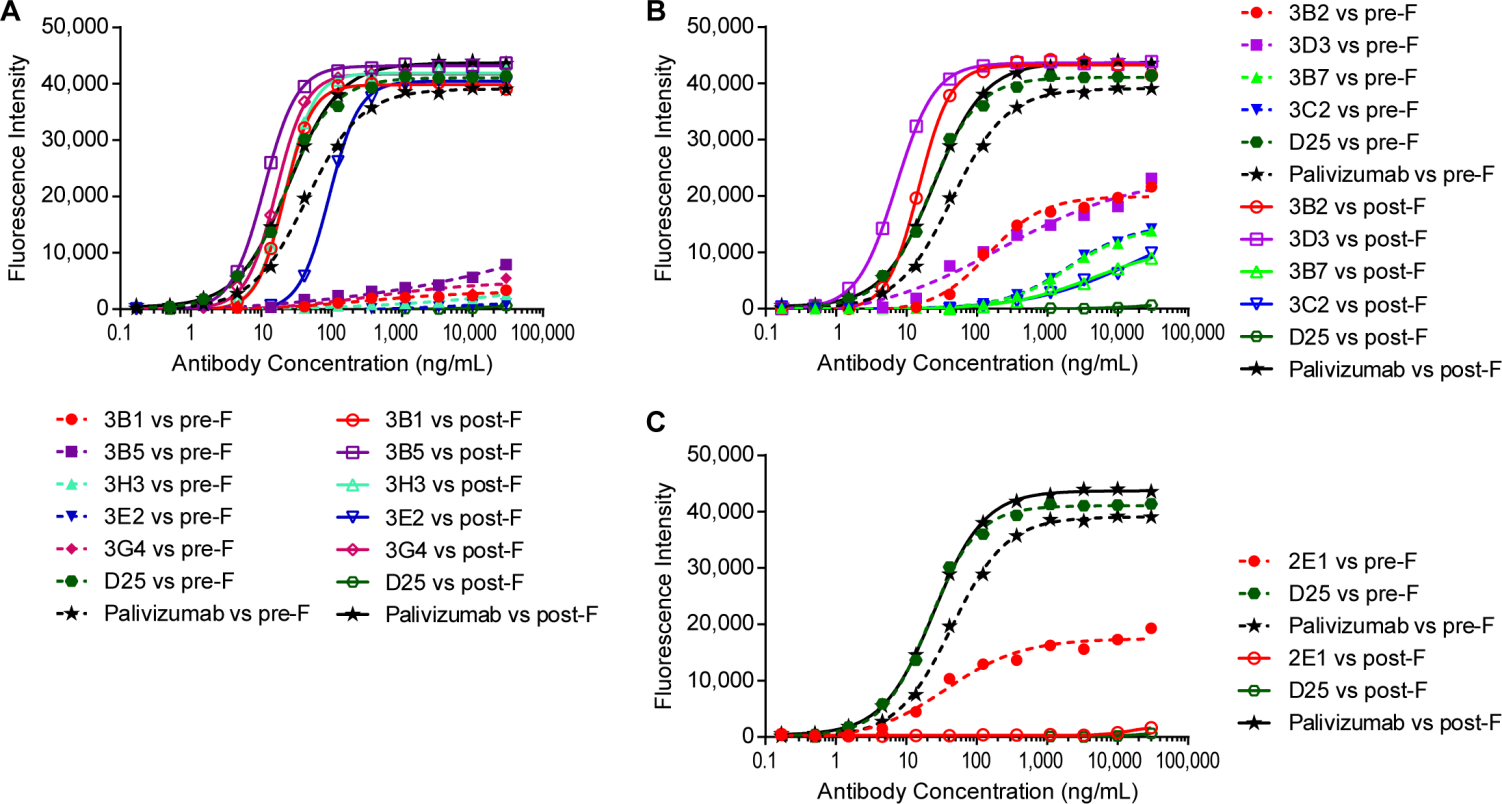
Anti-RSV F antibodies identified from Morphosys HuCAL GOLD^®^ phage display libraries. Heavy chain C-terminal 6xHis tagged antigen specific bivalent Fabs were purified with Ni-NTA column and then tested in ELISA binding to RSV prefusion and postfusion F proteins. (A) Antibodies preferentially binding to RSV postfusion F protein; (B) Antibodies binding to both RSV postfusion and prefusion F proteins; (C) antibody binding specifically to RSV prefusion F protein. Full-length human IgG1 D25 (prefusion F specific) and palivizumab (binding to both prefusion and postfusion F) were used as control antibodies in the above experiments.

For the generation of mAbs against the prefusion form of the F protein, three rounds of cross-panning were performed using prefusion F binding as positive selection and postfusion RSV F binding as negative selection. The inverse cross-panning approach was used in generating mAbs against postfusion F protein. Three groups of RSV F protein specific antibodies were identified from the Morphosys HuCAL GOLD^®^ phage display library (Fig 1): group 1 mAbs (3B1, 3B5, 3E2, 3H3, 3G4) which bind to the postfusion RSV F protein strongly with minimal binding to the prefusion F protein (Fig 1A); group 2 mAbs (3B2, 3D3, 3B7 and 3C2) which bind to both prefusion and postfusion F proteins (Fig 1B); and group 3 mAb (2E1) which binds specifically to prefusion F protein, although weakly as a bivalent Fab (Fig 1C).

**Fig 2:**
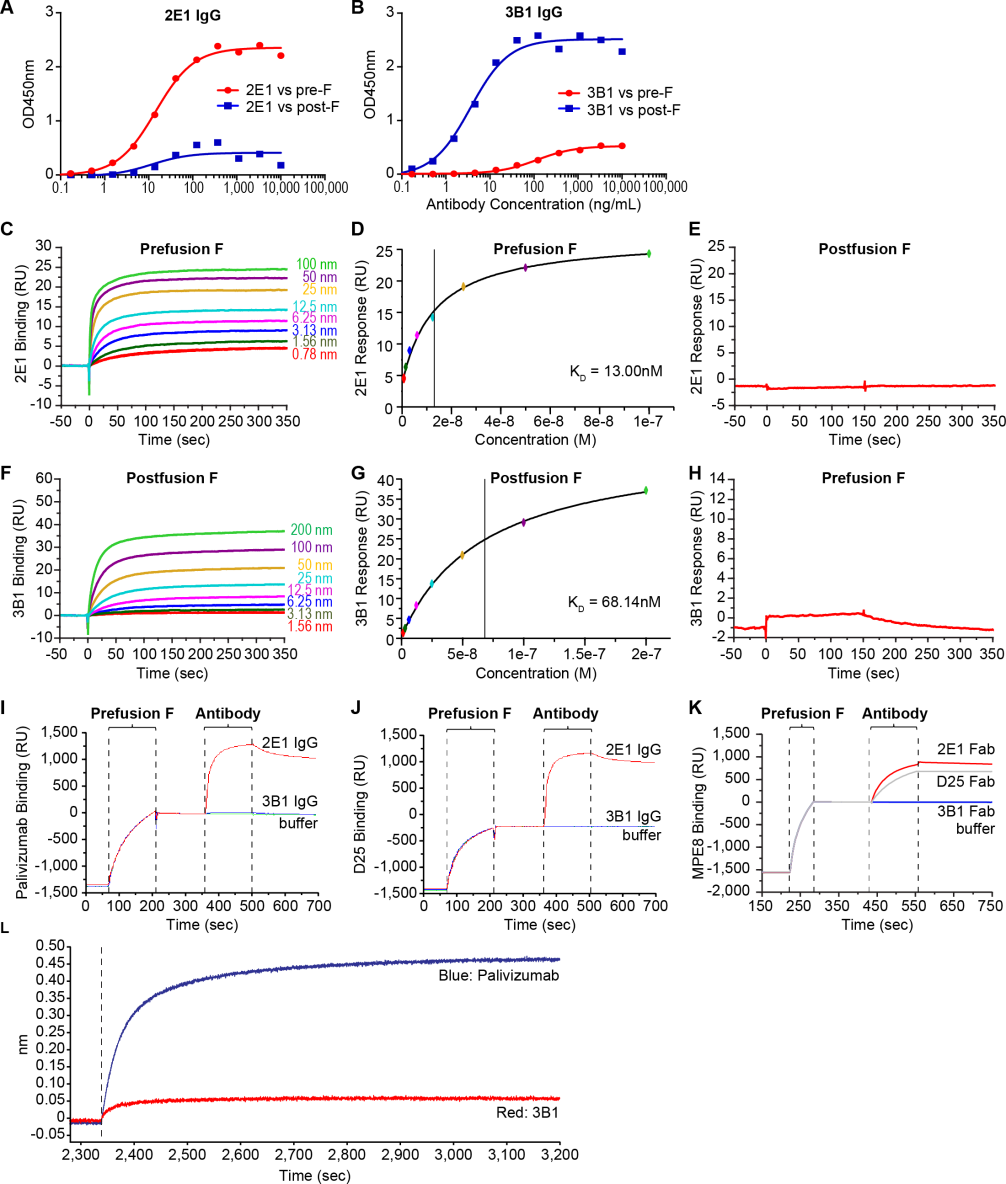
Characterization of mAbs 2E1 and 3B1. (A-B) ELISA analysis of 2E1 IgG (A) and 3B1 IgG (B) binding to RSV pre- (red circle) and postfusion F (blue square) proteins. (C-H) Surface plasmon resonance (SPR) analysis of 2E1 and 3B1 Fabs binding to pre- and postfusion RSV F proteins. RU = Resonance Units. Monovalent Fab antibody fragments were captured on the surface of a Series S Sensor Chip CM5 previously functionalized with Human Fab Binder. Prefusion or postfusion F protein diluted 2-fold serially starting at 100 nM or 200 nM was then injected over captured 2E1 (C) or 3B1 (F), respectively. To determine steady-state affinity, response levels at equilibrium were plotted over concentration of pre- (D) or postfusion F (G) protein. 50 nM of post- (E) or prefusion F (H) was injected to demonstrate the specificity of 2E1 binding to prefusion F and 3B1 to postfusion F. (I-K) SPR based competition analysis of 2E1 and 3B1 against palivizumab (I), D25 (J) and MPE8 (K) in binding to RSV prefusion F protein. RU = Resonance Units. Palivizumab and D25 were amine coupled to the surface of separate flow channels of a CM5 chip. A third flow channel was subjected to amine coupling activation without an antibody and used for reference subtraction. Prefusion F (40 μg/mL) was then injected over all surfaces. After a brief stabilization period, 2E1, 3B1, or running buffer was injected to measure binding to sites not occupied by the capturing antibody (palivizumab or D25). To assess competition to MPE8, the MPE8 antibody was captured (6000 RU, not shown) to flow channel 2 of a Biacore Sensor Chip Protein A. Prefusion F (40 μg/mL) was passed over channels 1 and 2 followed by 2E1 Fab, 3B1 Fab, D25 Fab and buffer to measure binding to sites not occupied by MPE8. (L) Bio-Layer Interferometry (BLI) based competition experiment of 3B1 IgG against site I antibody 131-2a. Palivizumab (blue) is able to bind to postfusion F protein in an Octet sandwich competition assay using 131-2a as the capture antibody, but antibody 3B1 (red) does not bind.

We converted the prefusion F specific bivalent Fab 2E1 into a full-length human IgG1 for further characterization because a weak binding bivalent Fab does not necessarily indicate that a full-length IgG version of the antibody will also be weak in its biological functions. We also converted 3B1 into a full-length IgG as a representative RSV postfusion F specific antibody because it worked well in binding exclusively to postfusion F protein as bivalent Fab. We showed that as full-length antibodies, both 2E1 and 3B1 bind strongly to their specific targets with EC50 values at 9.6 ng/mL and 3.6 ng/mL, respectively (Fig 2A and 2B). The sequences for the variable regions of 2E1 and 3B1 can be found in supporting information (S1 Table).

Surface plasmon resonance (SPR)-based studies demonstrated that 2E1 antibody binds exclusively to the prefusion conformation of the RSV F protein with an affinity (K_d_ = 13 nM for monovalent Fab, Fig 2C–2E). In contrast, 3B1 antibody binds only to the postfusion form of F, albeit with lower affinity (K_d_ = 68 nM for monovalent Fab, Fig 2F–2H). Competition studies demonstrated that mAb 2E1 recognizes an epitope outside the previously described sites 0, II and III, since its binding to prefusion F is not inhibited by palivizumab, D25 or MPE8 antibodies (Fig 2I–2K). Postfusion F specific mAb 3B1 competes binding to postfusion F with a previously identified site I antibody 131-2a [24], while palivizumab does not compete binding between postfusion F and 131-2a (Fig 2L), suggesting that 3B1 might recognize antigenic site I.

### 2E1 neutralized RSV potently

**Fig 3:**
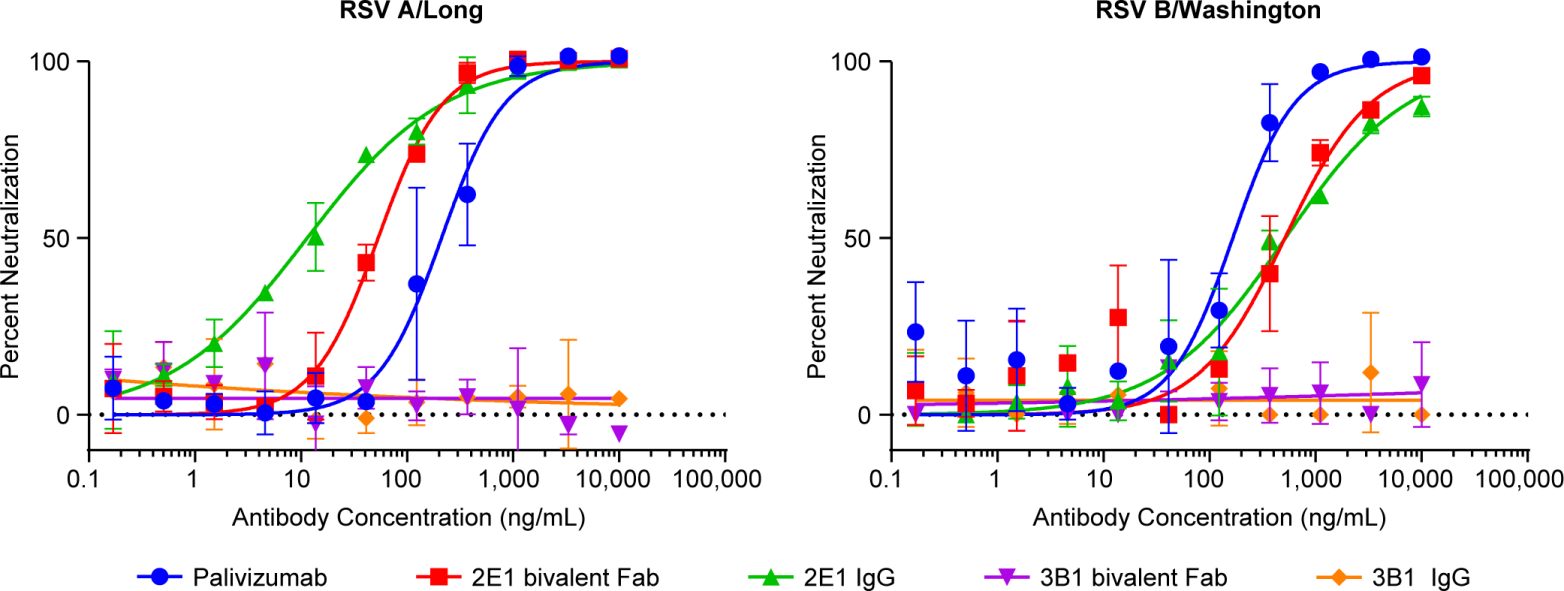
Neutralization of RSV by mAbs 2E1 and 3B1. Li-Cor based RSV micro-neutralization assay was performed. Neutralization curves against RSV A Long strain (A) and RSV B Washington strain (B) for 2E1, 3B1, and palivizumab are plotted with error bars representing the standard deviation from replicate data. 2E1 bivalent Fab is shown in red, full length IgG version of 2E1 shown in green, 3B1 bivalent Fab shown in purple, full length IgG version of 3B1 shown in orange, and palivizumab shown in blue.

All the bivalent Fabs generated in this study were tested in a Li-Cor based microneutralization assay. 2E1 bivalent Fab exhibits neutralizing activities against both RSV A (Long) and B (Washington) strains with IC50 at 53.4 ng/mL and 515 ng/mL, respectively (Fig 3). When converted into a full-length human IgG1, its neutralizing activity against the RSV A strain is greatly improved (IC50 at 11.4 ng/mL) and its IC50 against the RSV B (Washington) strain remained comparable (IC50 at 520 ng/mL) (Fig 3). Therefore, 2E1 appears to be an extremely potent neutralizing mAb against RSV A strain. Although its neutralizing activity against RSV B strain is much weaker than against A strain but is similar to that of palivizumab in the low-nanomolar range (Fig 3). In contrast, we did not detect any neutralizing activity with 3B1, either as bivalent Fab or full-length human IgG (Fig 3).

### Binding epitopes of 2E1 and 3B1 on RSV F protein were identified by shotgun mutagenesis

**Fig 4:**
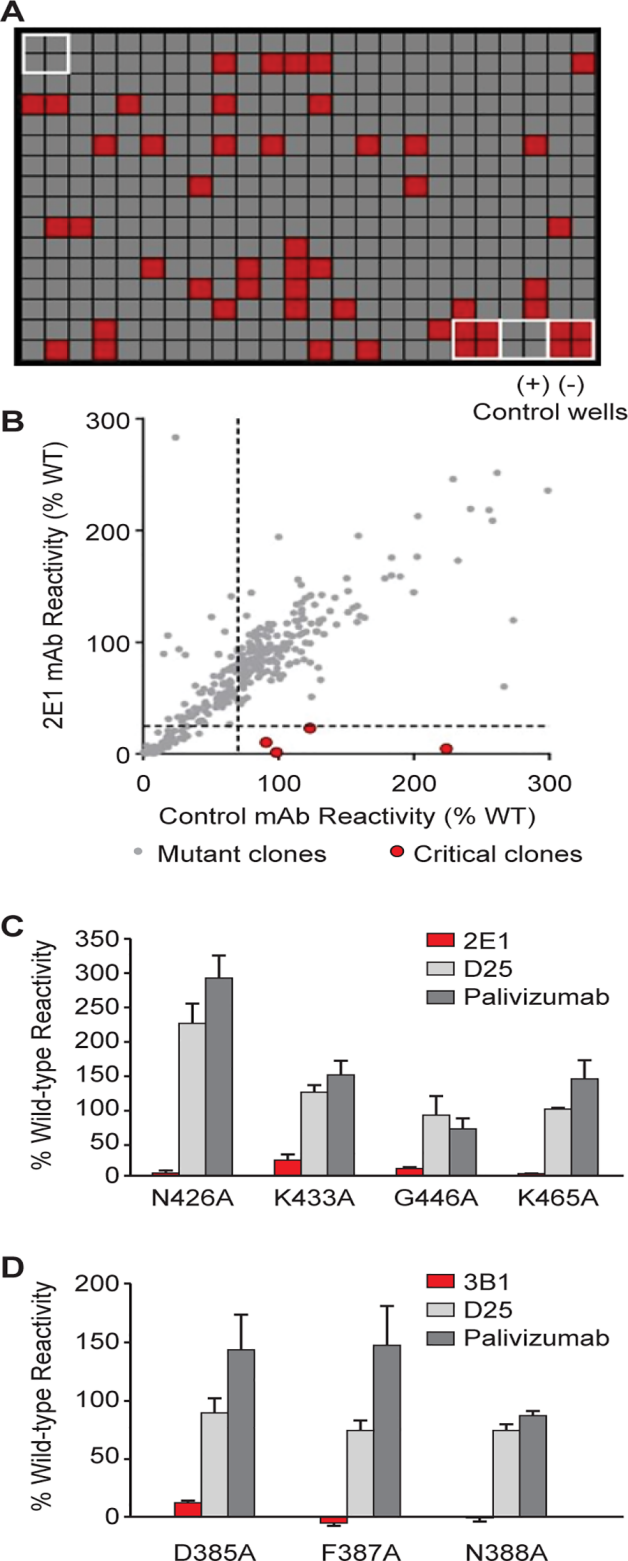
Identification of critical residues for mAbs 2E1 and 3B1 binding with shotgun mutagenesis epitope mapping. A shotgun mutagenesis alanine scanning library was constructed for the RSV F protein. The library contains 368 individual mutations at residues identified as surface exposed on the prefusion and postfusion forms of RSV F proteins. Each well of the mutation array plate contained one mutant with a defined substitution. (A) Reactivity results from a representative assay are shown with four positive (wildtype RSV F) and four negative (mock-transfected) control wells included on the plate. (B) Human HEK293T cells expressing the RSV F mutation library were tested for immunoreactivity with mAb 2E1, measured on an Intellicyt high-throughput flow cytometer. Clones with reactivity of <20% relative to that of wildtype RSV F (horizontal line) yet >70% reactivity for a control mAb (vertical line) were initially identified to be critical for mAb 2E1 binding (red dots), and were verified using algorithms described elsewhere [25]. (C) Mutation of four individual residues reduced 2E1 binding (red bars) but not the binding of D25 and palivizumab (gray bars). Error bars represent range (half of the maximum minus minimum values) of at least two replicate data points. (D) Mutation of three individual residues reduced 3B1 binding (red bars) but not the binding of D25 and palivizumab (gray bars). Error bars represent range of at least two replicate data points.

To map the binding epitopes of mAbs 2E1 and 3B1 on RSV F protein, an analysis was performed of the contact residues on the F protein using a shotgun mutagenesis methodology—a high-throughput cellular expression technology that enables the expression and analysis of large libraries of mutated target proteins within eukaryotic cells or on the cell surface [25]. 368 surface exposed residues were selected based on the crystal structures of the prefusion and postfusion RSV F proteins [10, 11] and 368 mutant expression constructs were generated as a comprehensive mutation library where every residue of interest was individually mutated to an alanine (and alanine to serine) for evaluation of loss of antibody binding (Fig 4A and 4B).

The RSV F mutation library was validated using previously characterized antibodies D25, which is specific for the prefusion conformation of RSV F, and palivizumab, which binds to both prefusion and postfusion F proteins. Critical residues are those amino acids whose side chains make the highest energetic contributions to the mAb-epitope interaction and the loss of antibody binding will be observed when these residues are mutated [26, 27]. By shotgun mutagenesis epitope mapping on RSV F, five critical residues for D25 binding (I64, Y198, L204A, V207, N208), and two critical residues for palivizumab binding (D269, K272) were identified, in agreement with the literature [11, 28]. Almost all these critical residues were previously shown to contribute to the epitopes for these antibodies [11, 29]. Interestingly, D25 bound well to cells expressing the wildtype RSV F sequence, suggesting that although the F protein did not include mutations to stabilize the protein in the prefusion conformation, there is still significant prefusion RSV F detected under our assay conditions, making the mutant library appropriate for mapping prefusion specific antibodies.

With this approach, four residues on the F protein were identified to be critical for binding to the prefusion specific mAb 2E1: N426, K433, G446, and K465. Alanine mutations at these positions significantly reduced 2E1 reactivity, while not affecting the binding of the control antibodies such as D25 and palivizumab (Fig 4B and 4C). These data suggest that the identified mutants are not misfolded or have lower surface expression levels, and that the identified residues are indeed directly involved in specific antibody binding. The data is also consistent with SPR analysis that 2E1 epitope is outside of site 0 or site II as 2E1 does not compete with antibodies specific to these sites. The low reactivity of N426A and K465A with mAb 2E1 suggests that N426 and K465 are the major energetic contributors to 2E1 binding (Fig 4C).

Similarly, three residues on the F protein were identified to be critical for binding to the postfusion-specific antibody 3B1: D385, F387 and N388 (Fig 4D). Alanine mutations at these positions significantly reduced 3B1 reactivity, while not affecting the binding of the control antibodies, suggesting that the identified mutants are not misfolded or have lower surface expression levels, and that they are directly involved in 3B1 binding. The low reactivity of F387A and N388A with 3B1 suggests that F387 and N388 are the major energetic contributors to antibody binding, with lesser contributions made by D385. Consistent with the antibody binding competition analysis (Fig 2L), the identified epitope is located in a short loop that contains the previously characterized antigenic site I of RSV F identified by a known escape mutant P389 [10, 30].

### Binding epitope of mAb 2E1 on prefusion RSV F protein was mapped by hydrogen/deuterium-exchange mass spectrometry

**Fig 5:**
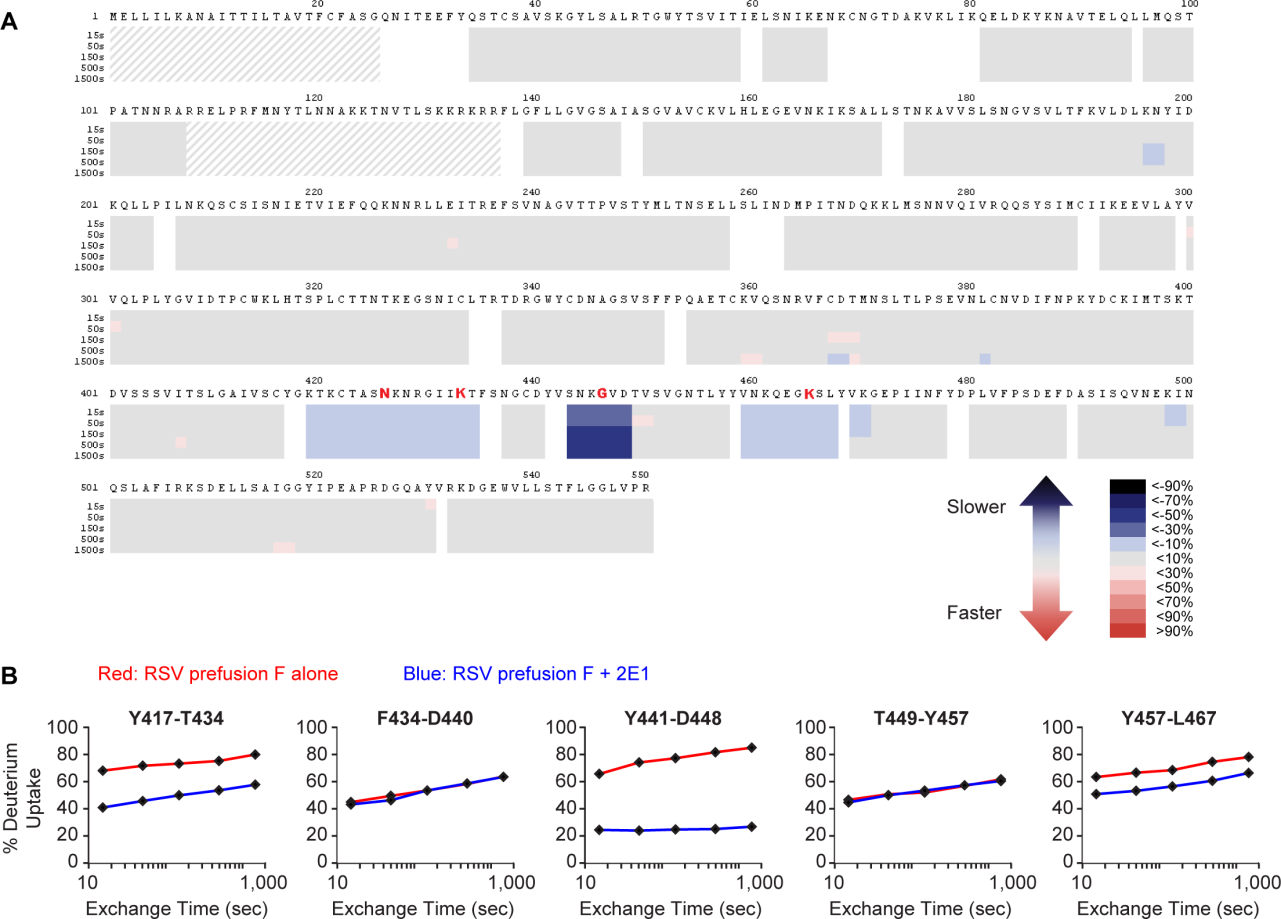
Epitope mapping of 2E1 by hydrogen/deuterium-exchange mass spectrometry. (A) Heat map plot showing the difference in deuteration levels of the RSV prefusion F protein alone compared to RSV prefusion F protein in the presence of the 2E1 monovalent Fab at five time points (15, 50, 150, 500, and 1500 sec). Slower deuterium exchange indicates regions containing the binding sites. White areas are ‘gaps’ for which there was no sequence coverage, and thus no HDX-MS information was obtained. Dashed greyed-out areas represent sequences of the signal and P27 peptides which are not present in the mature purified protein. (B) Uptake plots of several RSV F peptides spanning the conformational epitope region. Red curves show the deuteration levels of peptides of RSV F protein alone, while blue curves show the peptides of the RSV F / 2E1 complex. Peptides containing the residues of antibody epitope (417–434, 441–448, and 457–467) showed decreased deuteration level upon 2E1 binding. In contrast, peptides with no significant decrease in the deuteration level upon 2E1 exposure represent non-epitope sequences (435–440 and 449–457). The residues identified as critical for binding by shotgun mutagenesis are indicated in red font.

**Fig 6:**
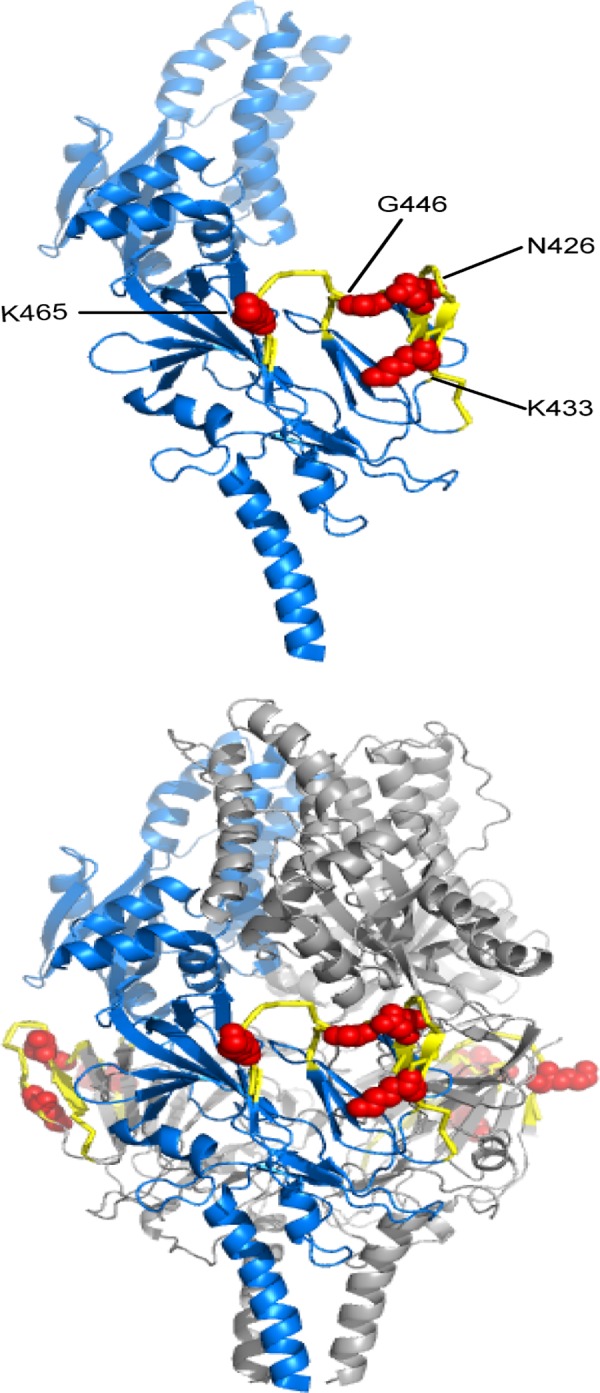
2E1 binding epitope visualized on the RSV F prefusion structure. The residues identified for 2E1 binding through shotgun mutagenesis are labeled and shown as red spheres and peptides identified through hydrogen/deuterium-exchange mass spectrometry are shown in yellow on the RSV F prefusion monomeric (top panel) and trimeric (bottom panel) structures [11].

Although only four amino acids on prefusion RSV F were identified as critical binding residues for 2E1 by Shotgun Mutagenesis, the actual epitope might involve more surface area. Shotgun mutagenesis might not be able to identify other residues that could also interact with the antibody if single alanine mutations at these positions are not enough to abolish the high affinity 2E1 binding. Therefore, we performed additional epitope mapping of 2E1 to prefusion RSV F protein using an in-solution hydrogen/deuterium exchange mass spectrometry (HDX-MS) analysis [31]. In this set of experiments, the deuteration levels of RSV F were monitored in the presence and absence of 2E1 monovalent Fab. We identified a discontinuous (conformational) epitope spanning across three distinct sections of the RSV F protein: residues 419–434, 443–448, and 459–467 (Fig 5). The actual peptides monitored for these protein sequence segments were Y417-T434, Y441-D448, and Y457-L467, respectively. During HDX-MS analysis, the deuterium to hydrogen back exchange rate at the first two N-terminal amino acids of any peptide is much faster than the time scale of the experiment. Hence, no protection information can be obtained for these amino acids [32]. All four critical residues (N426, K433, G446, and K465) for 2E1 binding identified by shotgun mutagenesis were contained in these three peptide stretches (Fig 6).

The most protected segment appeared to be the one containing residues 443–448 (Fig 5). However, 2E1 antibody binding was not reduced as much by a G446A mutation located in this segment, when compared to an N426A or K465A mutation (Fig 4). It is possible that a mutation to alanine from glycine is not as disruptive as a mutation from a polar/charged amino acid such as asparagine or lysine. In addition, this segment is only six amino acids long, while one of the segments with lower (but still significant) protection, 419–434, is sixteen amino acids long. The longer peptide likely contains many residues that are not involved in the antigen/antibody interaction and these residues might play a role in the reduction of observed deuteration level for the entire peptide. Therefore, there is a possibility that the specific residues contained within the epitope on the longer peptide are as strongly protected as those seen in the shorter peptide. The methods employed in these HDX-MS experiments only allow for the measurement of deuteration at a peptide level, not at a single amino acid level. As a result, the individual amino acids that make up the epitope are not identified, but rather the entire peptide which contains these residues.

Furthermore, segment 469–477 (peptide L467-I477) also showed protection above the baseline: about 5% difference in deuteration level between RSV F vs RSV F bound to 2E1, as compared to ~0 ± 1% baseline. This suggests that the actual epitope observed on segment 459–467 might be more towards the C-terminal section of the peptide and spans through the N-terminal portion of segment 469–477. This observation is consistent with the shotgun mutagenesis data which shows that K465 is one of the major energetic contributors to 2E1 binding (Fig 4).

## Discussion

We have isolated a panel of RSV F protein specific human monoclonal antibodies from Morphosys HuCAL GOLD^®^phage libraries by panning against RSV prefusion and postfusion F proteins and identified mAbs specifically against prefusion F and/or postfusion F proteins.

Among these antibodies, 2E1 is a prefusion F specific antibody while 3B1 is postfusion F specific. 2E1 showed potent neutralizing activity against both RSV A and B strains. Interestingly, in the primary bacterial lysate screening, 2E1 showed barely detectable binding to prefusion F protein in ELISA (data not shown). It also did not show strong binding as a purified bivalent Fab (Fig 1C). However, when we converted it into a full-length human IgG, 2E1 showed a potent binding activity specifically to the RSV prefusion F with an EC50 of 9.6 ng/mL (Fig 2A). The bivalent Fab was expressed in *E*. *coli* while the full-length IgG was produced in CHO cells, therefore, it is possible that appropriate post-translational modifications might help proper folding of the antibody and result in higher binding affinity.

Binding competition and shotgun mutagenesis epitope mapping studies suggest that 3B1 recognizes antigenic site I, the only known antigenic site which is postfusion specific. In addition, three secondary critical residues, R235, V243, and L467, were also identified as contributors to 3B1 binding in our shotgun mutagenesis mapping study (data not shown). These residues are not surface exposed in the postfusion RSV F structure and therefore most likely not contact residues in the 3B1 epitope [10]. We hypothesize that these residues might participate in the formation of the postfusion F structure, enabling binding of 3B1 to its epitope. In particular, in the postfusion (but not prefusion) F trimer structure, R235 residues form salt bridges with E232 residues on adjacent monomers [10, 11], likely contributing to the structural stability of the postfusion F trimer. Consistent with the above hypothesis, R235A mutation has almost no effect on binding to prefusion specific mAbs such as D25 and 2E1, but does affect binding to another postfusion specific antibody that we discovered (data not shown).

Competition studies also suggest that 2E1 does not recognize either antigenic site 0 or site III, two prefusion specific antigenic sites that were recently identified [11, 13]. We utilized two approaches to map the epitope of 2E1 on RSV prefusion F protein: shotgun mutagenesis and hydrogen/deuterium-exchange mass spectrometry and data from these two approaches agreed with each other very well (Fig 6). Our findings suggest that antibody 2E1 binds in the proximity of partially overlapping antigenic sites IV/V/VI on RSV F protein previously identified by Lopez *et al* [30]. 2E1 is distinct from antibodies against antigenic site IV, such as 101F, as site IV antibodies recognize both prefusion and postfusion conformations [12, 28, 33]. AM14, a prefusion specific antibody identified from human PBMC, appears to bind to the similar region as 2E1. Co-crystal structure as well as monoclonal antibody-resistant mutant (MARMs) study suggested that residue N426, one of the critical residues for 2E1 binding, is also one of the critical residues that contribute to AM14 binding [34]. However, K433 and K465, which are also critical for 2E1 binding, do not appear to be near the interaction interface between AM14 and RSV F protein. Moreover, the AM14 antibody resistant mutation study suggested that residues L160 and N183 from another protomer contribute to AM14 binding, making this antibody trimer-specific. We did not observe the loss of 2E1 antibody binding with alanine mutations in this region, nor did we identify any peptide from this region that was protected from deuterium exchange in the presence of 2E1. These data suggest that although 2E1 and AM14 target a similar area on prefusion RSV F, there exists a significant difference in their interactions with the antigen. Higher resolution structures will be required to further elucidate the similarity and difference in antigen recognition between these two antibodies.

2E1 neutralized RSV A Long strain more potently than RSV B Washington strain. The four residues that we identified from shotgun mutagenesis as critical for 2E1 binding are all conserved between these two strains. When comparing the three stretches of peptides that were identified from hydrogen/deuterium-exchange mass spectrometry, sequences of the first two peptides are 100% conserved between the two strains while there are two residues (462 and 466) which are different in the third peptide between the two strains. It is possible that these two residues contribute to the interactions with the antibody and changes in these positions result in changes in binding affinity and neutralizing activity. It is important to note that the prefusion F protein used for phage panning was derived from an RSV-A sequence, further arguing for the utility of the library in obtaining specific antibodies with fine specificities. When aligning the F sequences of RSV and human metapneumovirus (hMPV), three out of the four critical residues identified for 2E1 binding, except K433, were different in the hMPV F sequence. This is consistent with the observation that 2E1 is not able to neutralize hMPV (data not shown).

In this study, we successfully isolated human mAbs from Morphosys HuCAL GOLD^®^ phage libraries against RSV prefusion and postfusion F proteins. These antibodies have different functional specificities. The prefusion specific antibody 2E1 can efficiently neutralize both RSV A and B strains. The mAbs identified in this study will be useful as critical reagents in RSV vaccine and therapeutic development.

## Materials and Methods

### Preparation of RSV prefusion and postfusion F proteins

Plasmids encoding RSV F prefusion (DS-Cav1) and postfusion (F ΔFP) proteins based on strain A2 sequences with mammalian codon-optimization were used to transfect Expi 293F cells (Invitrogen) and proteins were purified as described previously [10, 11]. Briefly, RSV F proteins were purified from cell culture supernatants using Ni-Sepharose chromatography (GE healthcare). Postfusion RSV F was further purified by Strep-Tactin chromatography (Strep-Tactin Superflow Plus, Qiagen). Tags were removed by digestion with thrombin overnight. Prefusion F was further purified by a second Ni-Sepahrose chromatography step to remove IMAC contaminants and uncleaved RSV F. Both forms of RSV F proteins were further purified by gel filtration chromatography (Superdex 200, GE Healthcare).

### Phage display library panning

The human combinatorial antibody HuCAL GOLD^®^ libraries (Morphosys AG) were used in cross-panning against RSV prefusion F and postfusion F proteins following methods described by Krebs B [35]. Briefly, all 6 kappa and 6 lambda antibody phage libraries were mixed together to increase the chance of identifying antigen-specific antibodies. For the generation of mAbs against prefusion F protein, the mixed phage libraries were first absorbed in wells coated with postfusion F protein (negative selection). Unbound phages were then transferred to wells coated with prefusion F protein (positive selection). After extensive washing, phages binding to prefusion F protein were eluted with 20 mM DTT in 10 mM Tris/HCl pH 8.0 and amplified by infection and overnight culture of TG1 *E*. *coli*. The amplified phages were harvested and used in the second round of cross-panning with negative and positive selections. In total, three rounds of panning were performed. Similar cross-panning approach was used in generating mAbs against postfusion F protein.

### Subcloning, screening and identification of antigen-specific bivalent Fabs

After three rounds of cross-panning, the *E*.*coli* colonies grown on LB agar plates with 1% glucose and 34 μg/mL chloramphenicol (Teknova) were scraped off and resuspended in 2-YT broth (Teknova) with chloramphenicol. The bacterial pellets were harvested and proceeded for plasmid DNA preparation with QIAprep Spin Miniprep Kit (Qiagen). Minipreps of plasmid DNA were digested with EcoR I and Xba I, fragments encoding antibody sequences were agarose-gel purified with QIAquick Gel Extraction kit (Qiagen), and subcloned into Morphosys bivalent Fab expression vector pMORPHx9_Fab_dHLX_MH with Myc tag and His_6_ tags.

Constructs with antibody DNA fragment inserts were used to transform TG1F- electrocompetent cells which were then spread onto LB agar square plates with 1% glucose and 34 μg/mL chloramphenicol (Teknova). The plates were incubated at 37°C overnight. Each transformant was grown in 100 μL of 2-YT broth containing 1% Glucose and 34 μg/mL chloramphenicol in a master 96-well plate at 37°C overnight, with shaking at 250 rpm. 5 μL of overnight culture from each well of the master plate was inoculated into a 96-well expression plate containing 100 uL of 2-YT broth with 0.1% glucose and 34 ug/mL chloramphenicol per well. The expression plate was incubated at 37°C with shaking for 4–6 hrs. The bivalent Fab expression was induced by addition of 20 μL per well of 2-YT broth with 34 μg/mL chloramphenicol and 3 mM IPTG (at a final concentration of 0.5 mM IPTG). The expression plate was cultured overnight at 30°C with shaking.

Overnight induced *E*.*coli* cultures were first frozen at -70°C for 1 hr, then thawed under room temperature. Bacteria were then lysed by addition of 40 μL per well of BEL buffer (400 mM Boric Acid, 300 mM NaCl, 5 mM EDTA, 2.5 mg/mL Lysozyme) and shaking at 400 rpm for 1 hr at room temperature. 40 μL of 12.5% non-fat dry milk prepared in 1x TBS (Fisher Scientific) was added and incubated for 30 min at room temperature with shaking at 400 rpm. Samples were tested without dilution in ELISA assay described below for binding to prefusion and postfusion F proteins. Wells with fluorescence signal 2-fold higher than the negative control (no Fab lysate) were treated as positive hits and picked for sequence analysis. Unique antibody sequences were identified and corresponding bivalent Fabs were then produced.

### Production of bivalent Fab

Glycerol stock of E. coli carring vector expressing specific bivalentFab was inoculated into 20 mL of 2-YT broth containing 1% glucose and 34 ug/mL chloramphenicol and incubated overnight at 37°C. 7.5 mL of overnight culture was transferred to a 2L flask containing 400 mL of 2-YT broth with 34 ug/mL chloramphenicol and incubated at 37°C with shaking at 250 rpm for 4–6 hrs until the OD_600_ reaching 0.9–1.0. IPTG was then added to the culture at a concentration of 0.75 mM for induction, and the culture was incubated overnight at 30°C with shaking at 250 rpm. The overnight culture was centrifuged at 6000 rpm for 10 min at 4°C. The supernatant was discarded and pellet was frozen at -80°C.

To prepare lysis buffer, 1 tablet of Complete EDTA-free protease inhibitor cocktail (Roche) was added into 50 mL of NPI-10 buffer (50mM NaH_2_PO4, 300mM NaCl, 10mM imidazole, pH adjusted to 8.0 using NaOH). The frozen cell pellet was resuspended in 10 mL of lysis buffer and transferred to a centrifuge tube containing 1 mL of Lysozyme (Sigma) at 10 mg/mL and Benzonase (Novagen) at 3 units/mL of culture. The tube was incubated for 30 min at room temperature with shaking at 180–200 rpm and then centrifuged at 13,000 rpm for 10 min at 4°C. The clear lysate was purified with Ni-NTA column (Qiagen). Bivalent Fab containing C-terminal His_6_ tag was captured by Ni-NTA beads, and eluted with NPI-250 buffer (50mM NaH_2_PO4, 300mM NaCl, 250mM Imidazole, pH 8.0). The eluted bivalent Fab was buffer exchange with PBS (Hyclone). The concentration of bivalent Fab was determined with NanoDrop at 280 nm.

### Production of monovalent Fab

Monovalent Fab 2E1 and 3B1 were produced by Wacker Biotech GmbH (Jena, Germany) with its propertary *E*. *coli* expression system. Monovalent Fab with His_6_ tag was finally purified through Ni-NTA column.

### Conversion and production of full-length human IgG1

The heavy and light chain variable region sequences of antigen-specific bivalent Fabs were sub-cloned into pTT5 vector for CHO-3E7 cell expression. CHO-3E7 cells were grown in serum-free FreeStyle CHO Expression Medium (Life Technologies). The recombinant plasmids encoding heavy and light chains of each antibody were transiently co-transfected into 100 mL suspension CHO-3E7 cell cultures. The supernatants collected after 6 days were applied to Protein A CIP column (GenScript) for purification. The purified antibodies were buffer-exchanged to 1 x PBS, and QC tested by SDS-PAGE and Western blot analysis.

### ELISA

For testing bivalent Fabs, 96-well Maxisorp ELISA plates (Thermo Scientific) were coated with RSV prefusion or postfusion F protein overnight at 4°C. Threefold serially diluted antibodies were prepared in 1% nonfat milk/TBST, transferred to antigen coated plates, and incubated for 1 hr at RT with shaking at 150–200 rpm. Plates were washed 5 times with TBST. 100 μL per well of 1:40,000 diluted alkaline phosphatase-conjugated goat anti-human IgG ((Fab’)_2_ specific, Jackson ImmunResponse) was then added and incubate for 1 hr at room temperature with shaking at 150–200 rpm. Plates were washed 5 times with TBST. 100 μL of AttoPhos substrate was added to each well (1:5 diluted in TBST). After 4-7min incubation at RT, fluorescence signals were read with a Tecan F500 plate reader at 435 nm for excitation and 530 nm for emission. For testing full-length human IgGs, 1:2,000 diluted HRP-conjugated goat anti-human IgG (Southern Biotech) was used as secondary antibody. SuperBlu-Turbo TMB substrate (ViroLABS) was used as substrate for color development and plates were read at 450 nm.

### Surface plasmon resonance

Surface plasmon resonance (SPR) experiments were performed on a Biacore T200 (GE). A Series S Sensor Chip CM5 was functionalized with Human Fab Binder using the Human Fab Capture Kit (GE Healthcare). 2E1 or 3B1 monovalent Fab was captured (< 30 RU) on channel 4 and prefusion RSV F protein was injected in channels 3 and 4 at a flow rate of 40 μL/min for 360 sec at concentrations of 0.78, 1.6, 3.2, 6.4, 12.5, 25,50 and 100 nM, or postfusion RSV F protein at concentrations of 1.6, 3.2, 6.4, 12.5, 25, 50, 100, and 200 nM. Channel 2was used as reference control. Data were processed using Biacore T200 Evaluation Software and Kd values were calculated by fitting responses at equilibrium to a 1:1 model. For competition studies, palivizumab and D25 antibodies were covalently bound to different channel surfaces of a Sensor Chip CM5 through amine coupling at 2,000 resonance units (RU). A third channel surface with no antibody created under identical coupling conditions was used as reference. Prefusion F protein was injected at 40 μg/mL at a flow rate of 10 μL/min for 150 sec. After 150 sec dissociation, 2E1 or 3B1 antibody at 40 μg/mL was injected at 10 μL/min flow rate for 150 sec. To assess competition to MPE8, the MPE8 antibody was captured (6000 RU) to flow channel 2 of a Biacore Sensor Chip Protein A. Prefusion F (40 μg/mL) was passed over channels 1 and 2 at 10 μL/min followed by 2E1 Fab, 3B1 Fab, D25 Fab (40 μg/mL each) or buffer to measure binding to sites not occupied by MPE8. The data were processed with a BIAevaluation software with values from reference channel subtracted.

### Biolayer interferometry sandwich competition assays

Biolayer interferometry (BLI) assays were performed using an Octet Red 96 instrument (ForteBio, Inc.). Site I specific antibody 131-2a (5 μg/mL) was immobilized on anti-mouse Fc capture (AMC) biosensors. The biosensors were blocked with a non-specific isotype control IgG to saturate the binding sites, and then immersed into wells containing the antigen (post-fusion RSV F diluted to 4 μg/mL in kinetics buffer). Finally, the antigen loaded biosensors were immersed into wells containing either palivizumab or antibody 3B1(5 μg/mL) to measure competition. After each binding step of the assay, the biosensors were immersed in kinetics buffer and the baseline interference was read for 60 seconds in kinetics buffer (PBS, 0.01% BSA, 0.02% Tween 20, and 0.005% NaN_3_).

### RSV neutralization assay

Antibodies were diluted in EMEM containing 2% FBS (heated inactivated) starting at 10 ug/mL followed by 3-fold serial dilutions. 100 μL of diluted antibodies was mixed with equal volume (100 pfu) of RSV A (Long) strain or RSV B (Washington) strain and incubated for 1hr at 37°C. At the end of incubation, HEp-2 cells were added to the plates at 1.5x10^4^ cells/well. The plates were incubated at 37°C for 3 days. Afterwards, the cells were washed and fixed with ice-cold 80% acetone for 15 minutes. Mouse anti-RSV-F and anti-RSV-N mAbs (in-house generated, clone 143-F3-1B8 and 34C9, repectively) at 1.25 ug/mL each were added to the plates and incubated for 1 hr at RT. Plates were then washed and biotinylated goat anti-mouse IgG (Vector Laboratories) at 1:200 dilution was added. One hour later, the plates were washed and developed by a dual channel near infrared detection (NID) system (Licor Odyssey Sa). Infrared dye-Streptavidin to detect RSV specific signal and two cell stains for assay normalization were added to the 96-well plates. Plates were incubated for 1 hr in the dark, washed and dried in the dark for 20 minutes. Plates were then read on the Licor Aerius^®^ Automated Imaging System utilizing a 700 channel laser for cell normalization and an 800 channel laser for detection of RSV specific signal. 800/700 ratios were calculated and serum neutralizing titers were determined by four parameter curve fit in GraphPad Prism.

### Shotgun mutagenesis epitope mapping

Shotgun mutagenesis epitope mapping for antibodies 2E1 and 3B1 was performed by Integral Molecular, Inc. (Philadelphia, USA). Alanine scanning mutagenesis of an expression construct for RSV F (from RSV-A2; NCBI ref # FJ614814) targeted 368 surface-exposed residues identified from the crystal structures of both prefusion and postfusion conformations of RSV F [10, 11]. Each residue of interest was individually mutated to an alanine (and alanine residues to serine). The resulting 368 mutant RSV F expression constructs were sequence confirmed and arrayed into a 384-well plate (one mutation per well).

Library screening was performed essentially as described previously [36]. The RSV F alanine scan library clones were transfected individually into human HEK-293T cells and allowed to express for 16 hrs before fixing cells in 4% paraformaldehyde (Electron Microscopy Sciences) in PBS plus calcium and magnesium. Cells were incubated with mAbs diluted in 10% normal goat serum (NGS) for 1 hour at room temperature, followed by a 30 minute incubation with 3.75 μg/mL Alexa Fluor 488-conjugated secondary antibody (Jackson ImmunoResearch Laboratories) in 10% NGS. Primary mAb concentrations were determined using an independent immunofluorescence titration curve against wildtype RSV F to ensure that the signals were within the linear range of detection. Cells were washed twice with PBS without calcium or magnesium and resuspended in Cellstripper (Cellgro) plus 0.1% BSA (Sigma-Aldrich). Cellular fluorescence was detected using the Intellicyt high throughput flow cytometer (Intellicyt).

Antibody reactivity against each mutant clone was calculated relative to wildtype protein reactivity by subtracting the signal from mock-transfected controls and normalizing to the signal from wildtype protein-transfected controls. Mutations within clones were identified as critical to the mAb epitope if they did not support reactivity of the test mAb, but supported reactivity of other antibodies. This counter-screen strategy facilitates the exclusion of RSV F protein mutants that are misfolded or have an expression defect. The detailed algorithms used to interpret shotgun mutagenesis data are described elsewhere [25].

### Epitope mapping by in-solution HDX-MS

To optimize the sequence coverage, several preliminary experiments using various quench conditions, digestion, desalting, liquid chromatography, and mass spectrometry parameters were evaluated. Also, the optimal antigen-antibody ratio was determined in a separate titration experiment. The ratio was optimized by keeping a constant antigen concentration while changing the antibody concentration. Several peptides were identified whose deuteration levels decreased upon addition of the antibody. The optimal Ag:Ab ratio was identified when no additional decrease in deuteration levels were observed which represented equimolar amounts of Ag and Ab for binding. The following represents the conditions that were determined to be optimal for the hydrogen-deuterium exchange experiments. Stock solutions of 2.6 μM RSV F with and without an equimolar amount of 2E1 monovalent Fab were prepared in 1x PBS. The Ag:Ab complex was prepared by incubating RSV F with 2E1 at ambient temperature for 1 hr before cooling to 1°C. The hydrogen-deuterium exchange reactions were initiated by adding 40 μL PBS prepared in D_2_O to 10 μL of the RSV F and RSV F/2E1 solutions. The labeling reactions were performed at 1°C for five time points: 15, 50, 150, 500, and 5000 seconds. After these time points the samples were quenched by mixing 40 μL of the labeled solution with 20 μL of quench solution (2M Urea, 1M TCEP, adjusted to pH 3.0 with NaOH) at 1°C. 50 μL of the quenched sample was immediately injected onto an immobilized pepsin column (Waters Enzymate BEH Pepsin column, 2.1 x 30 mm) held at 10°C for on-line proteolysis using 0.05% (v/v) TFA in water solvent at a flow rate of 200 μL/min. The flow, with the digest, continued at the same rate onto a trap column (Waters UPLC BEH 300 C18, 1.7μm, 2.1 x 5cm) held at 0°C for desalting. The combined digestion and desalting steps lasted for 90 sec after which the trap column flow was reversed and eluted onto an analytical column (Waters Acquity UPLC CSH C18, 1.7μm, 1.0 x 100mm) held at 0°C using a gradient of solvent A: 0.05% (v/v) TFA in water and solvent B: 0.0025% (v/v) TFA in 95% acetonitrile / 5% water (v/v). A linear gradient of 13% to 40% B over 9.5 min at a flow rate of 40 μL/min was employed to elute the peptides into a ThermoScientific LTQ-XL Orbitrap mass spectrometer (Thermo Fisher Scientific) operated in positive mode at a resolution of 30,000. The data was acquired over the m/z range of 350–2000 in profile mode using the following MS parameters: HESI source voltage 4 kV, capillary voltage 44 V, tube lens 110 V, capillary temperature 250°C, and sheath gas flow 10. The labeling, quench, injection, digestion, desalting, and elution steps were performed using an HTC PAL robot (LEAP Technologies) controlled by HDxDirector software. The isocratic and gradient solvent flows were obtained using Waters nano-Acquity UPLC pumps (Waters). Prior to the exchange experiment, the identity of each peptide was confirmed by analyzing a non-deuterated RSV F sample in data-dependent MSMS mode and processed with Proteome Discoverer 1.4 software (Thermo Fisher Scientific). The HDExaminer software (Sierra Analytics) was used to determine the centroid mass of the isotopic envelope of each peptide in the labeling experiment and quantifying deuterium incorporation.

## Supporting Information

S1 Table Amino acid sequences of variable regions of mAb 2E1 and 3B1. (PDF)
