# Supplementary material for: Discovery and Characterization of Phage Display-Derived Human Monoclonal Antibodies against RSV F Glycoprotein
Source: PLoS One. 2016 Jun 3;11(6):e0156798. doi: 10.1371/journal.pone.0156798 (PMC4892554; doi:10.1371/journal.pone.0156798)
Supplement: S1 Table — (PDF) [file pone.0156798.s001.pdf]

S1 Table. Amino acid sequences of variable regions of mAb 2E1 and 3B1

	Heavy chain variable region	Light chain variable region
2E1	QVQLVESGGGLVQPGGSLRLSCAASGFTFSSYAITWVRQAPGKGLEWVSNINSDGSSTYYADSVKGRFTISRDNSKNTLYLQMNSLRAEDTAVYYCARTTFFMGLYFDVWGQGTLVTVSS	DIELTQPPSVSVAPGQTARISCSGDNIGSTYAYWYQQKPGQAPVLVIYDDTNRPSGIPERFSGSNSGNTATLTISGTQAEDEADYYCQTYDNQYLFGVFGGGTKLTVL
3B1	QVQLVQSGAEVKKPGSSVKVSCKASGGTFSDYYISWVRQAPGQGLEWMGGIIPIFGTANYAQKFQGRVTITADESTSTAYMELSSLRSEDTAVYYCARKYPVNFGGFASWGQGTLVTVSS	DIVMTQSPLSLPVTPGEPASISCRSSQSLLSSNGYTYLNWYLQKPGQSPQLLIYLGSSRASGVPDRFSGSGSGTDFTLKISRVEAEDVGVYYCQQYNNDPITFGQGTKVEIKRT
